# Effect of metalimnetic gradient on phytoplankton and zooplankton (Rotifera, Crustacea) communities in different trophic conditions

**DOI:** 10.1007/s10661-017-6055-7

**Published:** 2017-07-01

**Authors:** Maciej Karpowicz, Jolanta Ejsmont-Karabin

**Affiliations:** 1 Department of Hydrobiology, Institute of Biology, University of Białystok, Ciołkowskiego 1J, 15-245 Białystok, Poland; 2 Hydrobiological Station, Nencki Institute of Experimental Biology, Leśna 13, 11-730 Mikołajki, Poland

**Keywords:** Thermocline, Nutrients, Vertical distribution of plankton, Deep chlorophyll layer, Unpolluted lakes

## Abstract

Theory predicts and recent study revealed that depth of the thermocline can strongly influence the nutrient availability and composition of plankton communities. We are focused on the effect of metalimnetic gradients on water chemistry and plankton communities in three stratified lakes with different trophic conditions. Vertical changes in water chemistry revealed significant increase of macroelement concentrations in the metalimnion of all studied lakes. However, there was no significant increase of nutrient concentrations in the thermocline of lakes with smoother metalimnetic gradient, whereas sharp and deep thermocline zone caused higher concentration of orthophosphates and dissolved inorganic nitrogen in the metalimnion. The maximum concentrations of phytoplankton were observed just below the thermocline and were caused mostly by the abundance of diatoms and cryptophytes. Vertical distribution of the crustacean zooplankton was similar to the distribution of phytoplankton. Especially, *Daphnia cucullata* was strongly related with the phytoplankton distribution and reached maximum densities in deep layers with high chlorophyll concentrations, and, conversely, smaller crustacean species and rotifers were not affected by the vertical distribution of phytoplankton.

## Introduction

It is well known that metalimnetic gradients are key factors that influence nutrient cycling and structure of plankton communities in stratified lakes the during summer stagnation period (Gliwicz 1979; Cantin et al. 2011; Gauthier et al. 2014). During thermal stratification, nutrient depletion often occurs in the epilimnion of oligotrophic and mesotrophic lakes (Margalef 1983; Christensen et al. 1995; Padisak et al. 1998). Sharp vertical gradient of temperature in the metalimnion results in increased water density and viscosity, which decreases sinking speed of falling particles. These nutrient-rich waters of the metalimnion may play an important role in the functioning of phytoplankton in lakes of low trophy. Many previous studies revealed deep chlorophyll layers in thermally stratified lakes (e.g., Fee 1976; Barbiero and Tuchman 2004; Camacho 2006). The layers may be an important food source for zooplankton. The role of zooplankton is complex: they may enhance hypolimnetic algal growth by grazing in the epilimnion, which increases light penetration; but they may also feed on the hypolimnetic algal layer and reduce the biomass (Christensen et al. 1995). Finally, zooplankton could enriched the water with nutrients in a form available for phytoplankton. Phosphorus uptake by algae is directly affected by the rate of supply of available phosphorus forms, mostly by the orthophosphate phosphorus regenerated by the zooplankton rather than total phosphorus concentration (Ejsmont-Karabin and Spodniewska 1990).

The trophic state of a lake depends both on the intensity of nutrient loading from outside and on the rate of nutrient cycling within the lake ecosystem. Sharp metalimnetic gradients may cause the falling of particles which are trapped in the metalimnion. In lakes with sharp a thermocline, the symptoms of eutrophication may be more evident because of a decrease in water transparency in the epilimnion, whereas in lakes with a poorly defined thermocline, symptoms of eutrophication may be more visible in the hypolimnion due to an increase in the hypolimnetic oxygen consumption rate (Gliwicz 1979; Gliwicz and Kowalczewski 1981). Oxygen depletions could also prevent phosphorus loss from the epilimnion, because the orthophosphate phosphorus does not easily combined with metallic ions in reducing these conditions; thus, it is not carried down into the sediments (Gliwicz 1980).

The main goal of this study was to assess the effect of metalimnetic gradients on plankton communities in three stratified lakes in different trophic conditions. We predict that nutrient-rich waters of the metalimnion are preferred by phytoplankton and zooplankton communities. We analyzed the differences in concentration of orthophosphates, nitrogen forms (N-NH_4_^+^, N-NO_3_^−^, N-NO_2_^−^, DON, DN, PN, TN), and carbon forms (TOC, DOC, POC, IC, TC) in vertical profiles of the studied lakes. Furthermore, we expected that depth and shape of the thermocline could significantly influence water chemistry, plankton communities, and trophic state of lakes.

### Study sites

The three studied lakes (Hańcza, Szurpiły, and Jaczno) are located in the Suwalki Landscape Park (SLP) in the north-eastern part of Poland. The area represents a typical landscape formed during the last Vistulian glaciation and nearly 10% of the SLP is covered with postglacial lakes. The studied lakes have diverse morphology. Lake Hańcza is a typical deep channel lake which has a maximum depth of 108.5 m and is the deepest lake in Poland and in the Central European Lowlands. Lake Szurpiły and Lake Jaczno are typical deep moraine lakes with varied coastlines (Table 1) formed by the melting of dead ice blocks. The concentrations of nutrients in waters of the SLP lakes are much lower than in the other lakes of the region. Maintaining a low trophic level in the studied lakes is possible because of large buffering properties and low phosphorus loads from the catchment (Jekatierynczuk-Rudczyk et al. 2014). The catchments of the lakes are mostly unforested with extensive agriculture and population density less than 30 persons per square km.

**Table 1:** Morphometric and trophic characteristics of the studied lakes in Suwałki Landscape Park. Trophic status of lakes based on the present study and by Jekatierynczuk-Rudczyk et al. 2014

Lake	Latitude	Longitude	Surface of the lake (ha)	Maximum depth (m)	Average depth (m)	Length of coastline (m)	Shoreline development	SD visibility (m)	Trophic status
Hańcza	54^o^ 15, 9′	22^o^ 51, 1′	311.4	108.5	38.7	11,750	1.88	4.2	mesotrophic
Szurpiły	54^o^ 14, 3′	22^o^ 53, 5′	80.9	40	10	7000	2.19	2.9	meso-eutrophic
Jaczno	54^o^ 17, 0′	22^o^ 52, 3′	41.0	29.6	11.7	4840	2.13	2.9	eutrophic

The trophic status based on the biotic and abiotic parameters revealed differences between the studied lakes: Lake Hańcza is mesotrophic, Lake Szurpiły is meso-eutrophic (Jekatierynczuk-Rudczyk et al. 2014), and Lake Jaczno is eutrophic (Table 1). Detailed morphometric characteristics of the studied lakes were described by Borowiak et al. 2016. Hydrochemistry of the studied lakes was characterized by low orthophosphate content (Table 2). However, the lakes are clearly different as regards their electrical conductivity and concentration of oxygen in the hypolimnion zone (Table 2). The lowest values of electrical conductivity were observed in Lake Hańcza and the highest in Lake Jaczno (Table 2).

**Table 2:** The vertical variation of hydrochemical parameters in the studied lakes (mean values ± standard deviation)

	Hańcza			Szurpiły			Jaczno		
	E	M	H	E	M	H	E	M	H
Na^+^(mg l^−1^)	3.69 ± 0.63	*3.92 ± 0.68*	3.46	3.59 ± 1.86	3.98 ± 0.71	*4.29*	5.51 ± 0.09	5.6 ± 0.15	5.45
K^+^ (mg l^−1^)	2.11 ± 0.35	1.6 ± 0.79	2.23	1.40 ± 0.74	*1.87 ± 0.4*	1.71	1.60 ± 0.09	1.74 ± 0.08	1.75
Ca^2+^ (mg l^−1^)	44.6 ± 5.08	*54.78 ± 17.5*	40.09	44.6 ± 5.08	43.45 ± 1.5	*52.84*	56.6 ± 0.26	59.3 ± 2.2	*70.1*
Mg^2+^ (mg l^−1^)	11.1 ± 3.37	*13.1 ± 3.1*	8.47	10.39 ± 5.7	12.38 ± 3.5	14.2	17.9 ± 0.06	17.8 ± 0.11	17.58
SO_4_^2−^ (mg l^−1^)	10.95 ± 0.72	*11.14 ± 0.87*	10.42	5.8 ± 4.33	*10.85 ± 0.73*	11.05	12.28 ± 0.11	12.06 ± 0.21	9.53
Cl^−^ (mg l^−1^)	3.42 ± 0.25	3.27 ± 0.44	*3.80*	2.83 ± 0.87	*3.18 ± 0.32*	3.03	3.65 ± 0.06	*3.87 ± 0.09*	3.7
F^−^ (mg l^−1^)	0.18 ± 0.07	*0.23 ± 0.06*	0.14	0.17 ± 0.096	0.21 ± 0.06	0.239	0.31 ± 0.02	*0.35 ± 0.015*	0.31
N-NH_4_^+^ (mg l^−1^)	0.03 ± 0.02	0.034 ± 0.028	0.18	0.03 ± 0.03	0.034 ± 0.05	0.0187	*0.039 ± 0.023*	0.012 ± 0.004	0.008
N-NO_3_^−^ (mg l^−1^)	0.044 ± 0.07	0.01 ± 0.003	0.04	0.006 ± 0.002	*0.012 ± 0.01*	0.0048	0.009 ± 0.003	0.013 ± 0.004	*0.16*
N-NO_2_^−^ (mg l^−1^)	0.002 ± 0.001	0.001 ± 0.0005	0.00	0.001 ± 0.001	0.0015 ± 0.0004	0.0004	0.001 ± 0.0002	0.001 ± 0.0002	0.001
DIN (mg l^−1^)	*0.076 ± 0.07*	0.045 ± 0.026	0.04	0.037 ± 0.03	*0.047 ± 0.05*	0.024	0.049 ± 0.022	0.026 ± 0.006	*0.17*
DON (mg l^−1^)	0.26 ± 0.06	0.35 ± 0.02	0.36	*0.4 ± 0.13*	0.29 ± 0.06	0.306	0.12 ± 0.02	*0.16 ± 0.02*	0.028
DN (mg l^−1^)	0.33 ± 0.02	0.39 ± 0.02	0.40	*0.44 ± 0.12*	0.35 ± 0.004	0.33	0.16 ± 0.006	0.19 ± 0.015	0.20
PN (mg l^−1^)	0.079 ± 0.055	0.072 ± 0.03	0.09	*0.11 ± 0.06*	0.06 ± 0.004	0.056	0.005 ± 0.002	*0.016 ± 0.011*	0.01
TN (mg l^−1^)	0.41 ± 0.06	0.47 ± 0.05	0.48	*0.55 ± 0.18*	0.40 ± 0.008	0.387	0.17 ± 0.006	0.21 ± 0.014	0.21
PO_4_^3−^ (mg l^−1^)	0.023 ± 0.01	*0.033 ± 0.015*	0.02	0.019 ± 0.013	0.02 ± 0.003	0.0072	0.022 ± 0.008	0.017 ± 0.007	0.029
TOC (mg l^−1^)	*5.12 ± 0.26*	4.83 ± 0.1	4.53	4.4 ± 0.1	*4.59 ± 0.35*	3.73	2.62 ± 0.1	*2.93 ± 0.35*	2.81
DOC (mg l^−1^)	*4.87 ± 0.31*	4.6 ± 0.14	4.32	4.08 ± 0.04	*4.16 ± 0.25*	3.55	2.55 ± 0.04	*2.87 ± 0.39*	2.46
POC (mg l^−1^)	*0.25 ± 0.22*	0.22 ± 0.19	0.21	0.32 ± 0.1	*0.42 ± 0.15*	0.18	*0.068 ± 0.07*	0.052 ± 0.05	0.34
IC (mg l^−1^)	17.4 ± 0.44	17.97 ± 0.59	*18.64*	22.58 ± 0.49	23.57 ± 0.68	*26.33*	28.98 ± 0.87	31.27 ± 1.32	*38.09*
TC (mg l^−1^)	22.52 ± 0.41	22.8 ± 0.53	23.17	26.98 ± 0.44	28.17 ± 0.44	*30.06*	31.6 ± 0.88	34.2 ± 1.54	*40.9*

Values in italics are the highest values in the lakes’ profiles at *p* < 0.05

## Methods

The study was conducted in the peak of the summer stagnation (22–23 July 2015). The sampling stations were located close to the deepest point of Lake Hańcza and Lake Szurpiły. Lake Jaczno was sampled in the central basin with maximum depth of 16 m. Water samples for chemical analyses and zooplankton samples were taken every meter from the surface to the hypolimnion zone (0–11 m) by the 5 L Limnos sampler. Additionally, one sample from the center of the hypolimnion was taken.

The field measurements included the Secchi disc visibility, conductivity and concentration of dissolved oxygen by the Hach Lange Sonde. Phytoplankton communities (green algae, cyanobacteria, diatoms, cryptophytes, total chlorophyll *a* concentration) and temperature were measured by the submersible spectrofluorometer (FluoroProbe, bbe-Moldaenke). Constant measurements of temperature every few centimeters of depth allow us to determinate precisely the thermocline (metalimnion) zone. The FluoroProbe spectrofluorometer provides in situ measurements of total chlorophyll *a* and also algae classes determination using differences among fluorescence excitation spectra. Changes in the resulting chl *a* emission allow for fluorometric estimation of algal classes based on differences in species and class-dependent peripheral antenna pigments (Beutler et al. 2002). The FluoroProbe identifies the four phytoplankton classes: green algae (Chlorophyta and Euglenophyta), cyanobacteria (phycocyanin-rich cyanobacteria), diatoms (Heterokontophyta, Haptophyta, and Dinophyta) and cryptophytes (Cryptophyta and the phycoerythrin-rich cyanobacteria).

Five-liter zooplankton samples were taken from each meter, then filtered through a 50-μm plankton net and fixed with 4% formalin. Rotifers and crustaceans were determined to species and counted in the whole samples. Additionally, 10-length measurements were made for each species. The animal length was used to estimate the dry weight of crustaceans by applying the equations after (Błędzki and Rybak 2016). The biomass of rotifers was established following Ejsmont-Karabin (1998).

Analyses of chemical parameters of water were conducted immediately after sample collection in the laboratory. The concentrations of ions (PO_4_^3−^, N-NH_4_^+^, N-NO_3_^−^, N-NO_2_^−^, Ca^2+^, Mg^2+^, Na^+^, K^+^, SO_4_^2−^, Cl^−^, F^−^) were determined in a Dionex ICS 1100 ion chromatograph provided with an IonPac As-HC column, using a solution of 9 mM of Na_2_CO_3_ as eluent, with a flowrate of 1 mL/min and a pressure of around 2000 psi, based on Standard Methods 4110B (APHA 1998). Dissolved nitrogen (DN) was calculated as the sum of N-NH_4_^+^, N-NO_3_^−^, and N-NO_2_^−^. The concentrations of dissolved nitrogen (DN), total nitrogen (TN), total organic carbon (TOC), dissolved organic carbon (DOC), and inorganic carbon (IC) were analyzed by the high-temperature catalytic combustion in Shimadzu TOC-L Series analyzers. Particular nitrogen (PN) was calculated from the differences between TN and DN. Particulate organic carbon (POC) was calculated from the differences between TOC and DOC. Total carbon (TC) was calculated as the sum of TOC and IC (Cudowski et al. 2015).

The differences between analyzed variables were tested with the non-parametric Kruskal-Wallis test (*p* < 0.05). The agglomerative hierarchical classification (AHC) based on the Bray-Curtis similarity matrix was used to visualize the differences in water chemistry in vertical profiles of the studied lakes. The relations between the abundance of dominant zooplankton species to the vertical environmental variables (hydrochemistry and phytoplankton) in the studied lakes were visualized by the Canonical Correspondence Analysis (CCA). CCA is very useful tool for ecologists to relate the abundance of species to environmental variables (ter Braak 1986). Statistical analyses were performed with XLSTAT 2013 (Addinsoft).

## Results

### Hydrochemical gradients

**Fig. 1:**
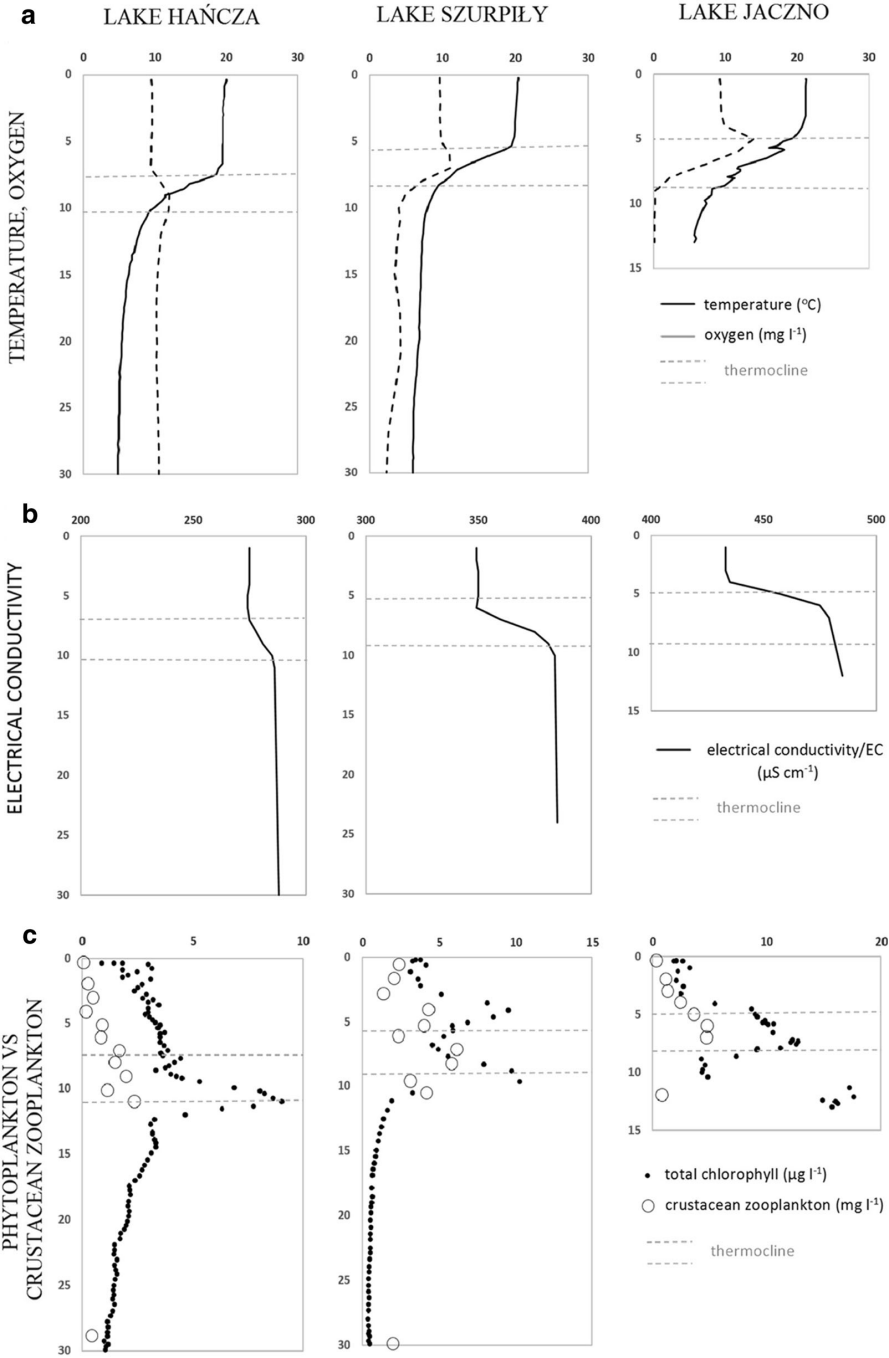
Vertical profiles of temperature and oxygen concentration (**a**), electrical conductivity (**b**), biomass of phytoplankton and zooplankton (**c**) in the studied lakes

The differences were observed in vertical gradients of water temperature in the studied lakes. Lake Hańcza was characterized by sharp temperature gradient and the greatest depth of the thermocline zone (Fig. 1a). Smoother temperature gradients were observed in Lake Szurpiły and Lake Jaczno (Fig. 1a). Oxygen concentrations were high in the epilimnion zones of all studied lakes; however, the maximum concentrations of oxygen were observed in the upper part of the metalimnion (Fig. 1a). Lake Hańcza has well-oxygenated hypolimnion with saturation above 100%, oxygen saturation of the hypolimnion in Lake Szurpiły ranged from 30 to 40%, while in Lake Jaczno was below 1%. The lakes clearly differed by electrical conductivity (EC). The lowest values of EC were observed in Lake Hańcza and the highest in Lake Jaczno (Table 2). Vertical changes of EC revealed significant increase in the metalimnion zone. The increase of conductivity in the metalimnion of Lake Hańcza was at about 5%, while in Lake Szurpiły about 9.5%, and in Lake Jaczno approximately 11.5% (Fig. 1b). Generally higher concentrations of macroelements (Ca^2+^, Mg^2+^, Na^+^, K^+^, SO_4_^2−^, Cl^−^) were found in the metalimnion and hypolimnion zones (Table 2). The studied lakes were characterized by low orthophosphate content. Lake Hańcza had the highest concentration of orthophosphate in the metalimnion, while in Lake Szurpiły the concentrations of orthophosphate were higher in the upper water layers than in the hypolimnion zone. There were no significant differences in vertical concentrations of orthophosphate in Lake Jaczno (Table 2). The vertical differences of organic and inorganic carbon concentrations were found in all lakes. Inorganic carbon (IC) had higher concentrations in the hypolimnion zones, while total organic carbon (TOC) had higher concentrations in the epilimnion and metalimnion (Table 2). The analyzed lakes differed in the concentrations of nitrogen forms in the vertical profiles. Only ammonium ions were found in higher concentrations in the upper water layers of all lakes (Table 2). Higher concentrations of dissolved inorganic nitrogen were found in the deep water zone of Lake Szurpiły and Jaczno, while in Lake Hańcza the highest DIN concentration was found in the epilimnion.

**Fig. 2:**
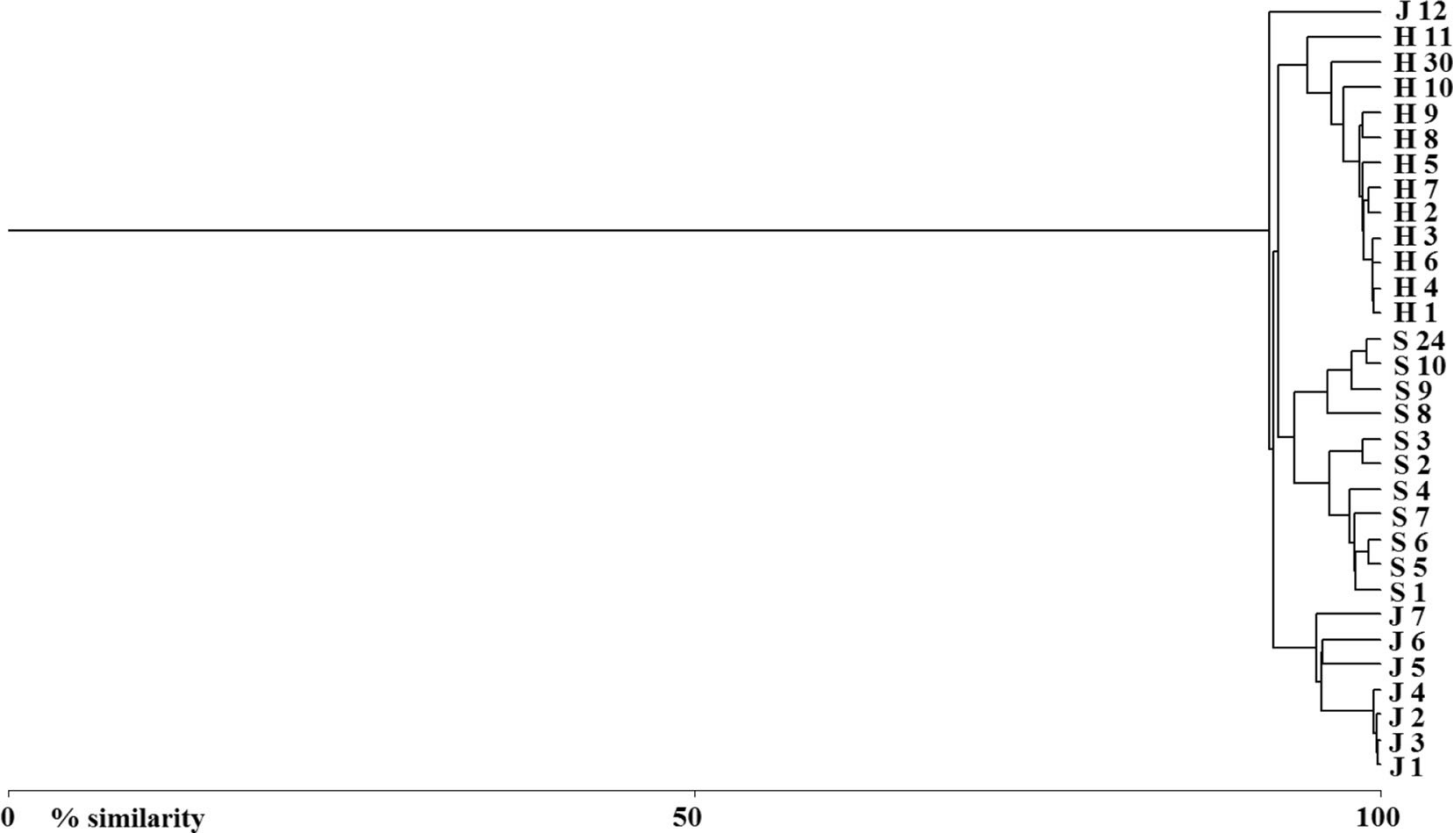
The Bray-Curtis similarity matrix of hydrochemical parameters in vertical profiles of Lake Hańcza (*H*), Lake Szurpiły (*S*), and Lake Jaczno (*J*) based on the agglomerative hierarchical cluster (AHC). The numbers behind the symbol of the lake is depth (m) of hydrochemical samples

The agglomerative hierarchical classification of similarity of hydrochemical parameters in vertical profile distinctly divided the studied lakes (Fig. 2). Very high similarity of hydrochemical parameters was noted in the epilimnion zones of the studied lakes, whereas these parameters in the deep water zone of Lake Hańcza and Szurpiły were very similar to the thermocline zone (Fig. 2).

### Vertical distribution of phytoplankton

Total chlorophyll *a* concentration in the studied lakes ranged between 0.88 and 17.62 μg l^−1^. The maximum concentrations of phytoplankton were observed in the upper part of the hypolimnion and in thermocline zone (Fig. 1c). In Lake Szurpiły and Jaczno, there were observed two significant increases of phytoplankton caused by different groups of algae (Fig. 2). The first increase of phytoplankton concentration in Lake Szurpiły was recorded in the lower epilimnion zone and was caused by diatoms and green algae. The second increase of phytoplankton in the upper hypolimnion zone of Lake Szurpiły was caused by diatoms and cryptophytes (Fig. 2). In Lake Jaczno, first significant increase of phytoplankton was observed in the metalimnion zone and was caused by diatoms and green algae, while the maximum concentration of phytoplankton in the upper hypolimnion was caused mostly by cryptophytes (Fig. 2). Often different groups of algae reached maximum density at different depths. Maximum concentrations of diatoms were found in the lower thermocline zone, while cryptophytes were reaching the highest density at greater depth. Green algae reached the highest densities in the epilimnion zone (Lake Szurpiły and Hańcza) and in the thermocline zone of Lake Jaczno (Fig. 2). Cyanobacteria were minor component of phytoplankton in the studied lakes.

### Vertical distribution of crustacean zooplankton

**Fig. 3:**
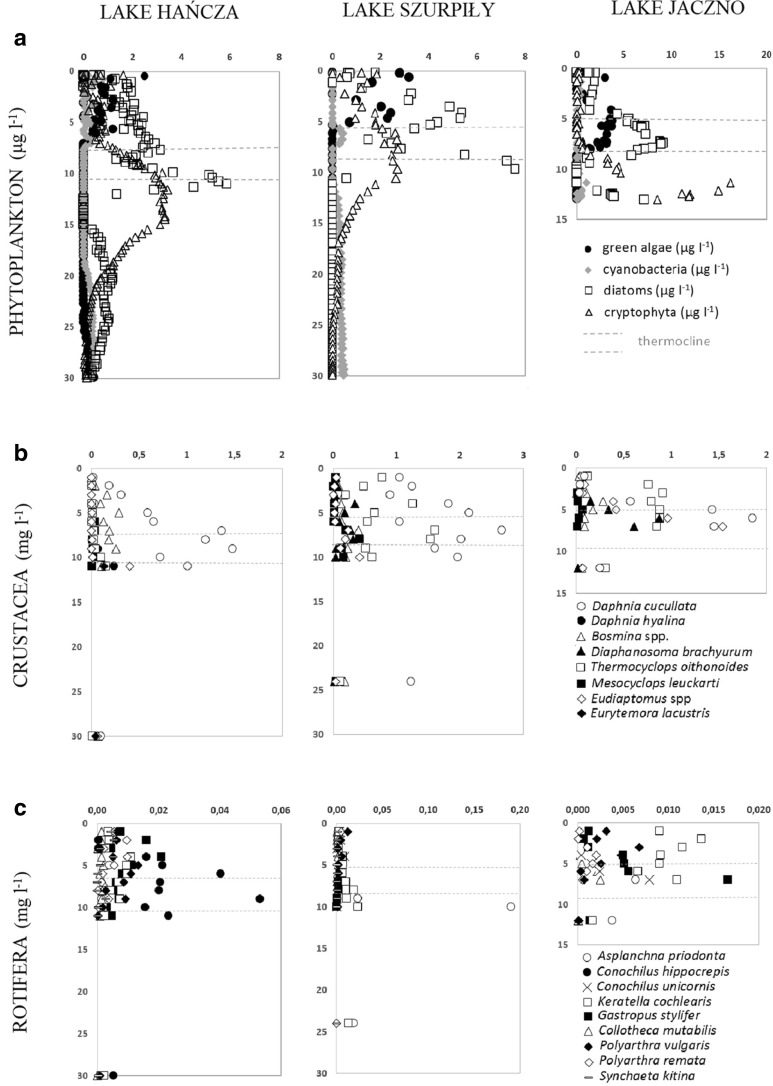
Vertical distribution of dominant phytoplankton groups (**a**), crustacean species (**b**), and Rotifera species (**c**) in the studied lakes

Maximum concentrations of crustacean zooplankton were found in the thermocline zones of the studied lakes. Vertical distribution of crustacean zooplankton was similar to the phytoplankton distribution (Fig. 1c). Especially in Lake Szurpiły, there were observed two significant increases of crustacean biomass which are similar with the occurrence of phytoplankton (Fig. 1c). The dominant crustacean species was *Daphnia cucullata*, reaching up to 80% in the total biomass of the crustacean zooplankton in Lake Hańcza and up to 66% in Lake Szurpiły. *D. cucullata* reaches maximum occurrence within the thermocline zones of the studied lakes (Fig. 3b), which is very similar with the maximum occurrence of phytoplankton (Fig. 3a). Few other crustacean species reached the highest biomass in the thermocline zone, i.e., *Thermocyclops oithonoides* in Lake Szurpiły and *Eudiaptomus* spp., *Diaphanosoma brachyurum*, and *T. oithonoides* in Lake Jaczno (Fig. 3b). Vertical distribution of the remaining crustacean species showed no significant differences in the lakes’ profiles.

### Vertical distribution of Rotifera

The biomass of the rotifer species was more or less segregated vertically in Lake Hańcza. In the upper part of the epilimnion, species prevailed in feeding on dinoflagellates (*Gastropus stylifer* Imhof, 1891), or smaller algae (*Polyarthra* spp), whereas in deeper part of the zone there was an observed increased of biomass of colonial *Conochilus hippocrepis* (Schrank, 1803)*. C. hippocrepis* decided also on a strong increase of rotifer biomass in the metalimnion (Fig. 3c).

A different distribution of rotifer species was noted in Lake Szurpiły (Fig. 3c). The highest biomass of rotifers was found below the metalimnion zone. However, it was built mostly by a large predatory species, *Asplanchna priodonta* Gosse, 1850. The biomass of the remaining smaller rotifers was similar at different depths, with *G. stylifer* and *Polyarthra* spp. dominating in the epilimnion, and *Keratella cochlearis* (Gosse, 1851) dominating in and below the metalimnion.

Domination of *K. cochlearis* and *Polyarthra vulgaris* Carlin, 1943 was observed also in the epilimnion of Lake Jaczno. At 4-m depth this community was joined by *G. stylifer*, which achieved the highest biomass in the middle layer of the metalimnion zone. The biomass of rotifers was similar at all depths down to the sixth meter and doubled at the seventh meter.

### Environmental factors affecting the vertical distribution of zooplankton

**Fig. 4:**
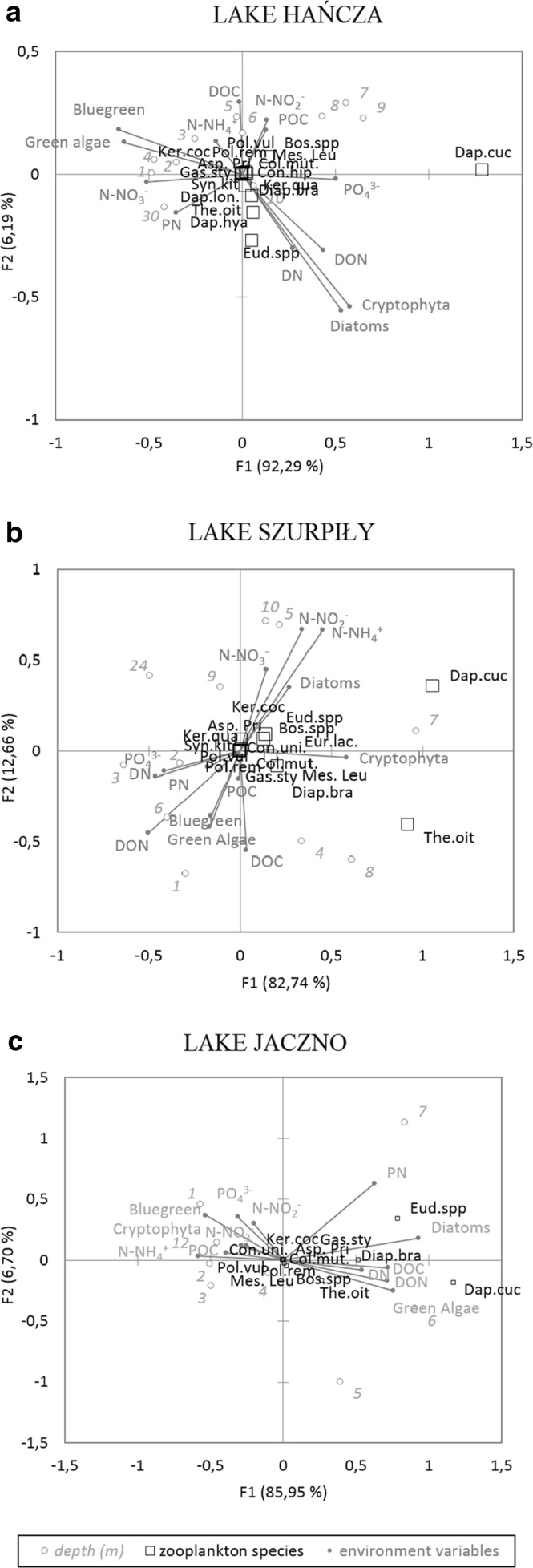
Relations between abundance of dominant zooplankton species to the vertical environmental variables (hydrochemistry and phytoplankton) in Lake Hańcza (**a**), Szurpiły (**b**), Jaczno (**c**) visualize by the Canonical Correspondence Analysis map. The taxa shown are Dap.cuc. – *Daphnia cucullata*, Dap.lon – *Daphnia longispina*, Dia.bra – *Diaphanosoma brachyurum, bos.spp. – Bosmina* species, Eud.spp. – *Eudiaptomus* species, Eur.lac – *Eurytemora lacustris*, The.oit – *Thermocyclops oithonoides*, Mes.leu – *Mesocyclops leuckarti*, Asp.pri – *Asplanchna priodonta*, Col.mut – *Collotheca mutabilis*, Con.hip – *Conochilus hippocrepis*, Con.uni – *Conochilus unicornis*, Gas.sty – *Gastropus stylifer*, Ker.coc – *Keratella cochlearis*, Ker.qua – *Keratella quadrata*, Pol.rem – *Polyarthra remata*, Pol.vul – *Polyarthra vulgaris*, Syn.kit – *Synchaeta kitina*

First axes of the CCA ordinations were the most important in the explained distribution of dominant zooplankton species in the vertical profile of the studied lakes (Fig. 4). Correlation values of environmental variables and sites (depth) with the first axes are shown in Table 3. Most of the samples from the thermocline in all lakes were positively correlated with the first axis of the CCA map. Dominant rotifer species were generally less affected by the environmental conditions than the Crustacea species in the studied lakes (Fig. 4). *D. cucullata* was strongly related to the phytoplankton distribution and water chemistry. Abundance of *D. cucullata* was associated with cryptophytes and diatoms in Lake Hańcza (Fig. 4a) and Lake Szurpiły (Fig. 4b). In Lake Jaczno, vertical distribution of *D. cucullata* and *Eudiaptomus* species was associated with diatoms, green algae, particulate nitrogen, dissolved organic carbon, and dissolved organic nitrogen (Fig. 4c). Abundance of *T. oithonoides* in Lake Szurpiły could be related to the abundance of diatoms and cryptophytes (Fig. 4b).

**Table 3:** Correlation values of environmental variables and sites (depth) with the first axes of CCA analysis (Fig. 4)

Environmental variables	Hańcza	Szurpiły	Jaczno	Sites (depth)	Hańcza	Szurpiły	Jaczno
N-NH_4_^+^	−0.14	0.45	−0.58	1	−0.48	−0.30	−0.57
N-NO_3_^−^	−0.51	0.15	−0.25	2	−0.35	−0.33	−0.50
N-NO_2_^−^	0.13	0.34	−0.20	3	−0.25	−0.63	−0.49
DON	0.44	−0.50	0.72	4	−0.47	0.33	−0.10
DN	0.27	−0.41	0.54	5	−0.02	0.21	0.39
PN	−0.35	−0.46	0.63	6	0.01	−0.39	0.92
PO_4_^3−^	0.50	−0.18	−0.31	7	0.55	0.96	0.83
DOC	−0.01	0.03	0.72	8	0.42	0.61	
POC	0.13	−0.01	−0.39	9	0.65	−0.10	
Green algae	−0.63	−0.16	0.76	10	0.06	0.14	
Cyanobacteria	−0.66	−0.16	−0.53	11	0.30		−0.45
Diatoms	0.53	0.27	0.93	25	−0.41	−0.49	
Cryptophytes	0.58	0.58	−0.29				

## Discussion

Our study confirms significant change of hydrochemical parameters in the metalimnion zone of the studied lakes. There was a significant increase of conductivity and concentration of macroelements in the metalimnion zone. Most of the studies describe the thermocline as a nutrient-rich layer (Fee et al. 1996; Wetzel 2001) but there was no significant increase of nutrient concentrations in the metalimnion of Lake Szurpiły and Jaczno. This could be caused by smoother metalimnetic gradients and by the high concentration of algae in this layer. The sharp and deep thermocline in Lake Hańcza was related to the highest concentration of orthophosphates in the metalimnion and high concentration of dissolved inorganic nitrogen in the epilimnion. The large depth of the thermocline makes particles to remain longer in the epilimnetic-mixed layer (Diehl 2002; Berger et al. 2006), and sharp metalimnetic gradients caused some falling particles to be trapped in the metalimnion (Gliwicz 1979; Gliwicz and Kowalczewski 1981). As a result, there was high oxygen concentration in the whole hypolimnion of Lake Hańcza. Deeper vertical mixing of water column in Lake Hańcza can also contribute to greater oxygenation of the hypolimnion (Scully et al. 2000). High similarity of hydrochemical parameters between the metalimnion and hypolimnion in Lake Hańcza and Szurpiły suggests that these layers could be mixed due to small difference in water density. Smoother thermocline gradient in Lake Jaczno results in faster sinking speed of particles and as a consequence, there was significantly higher nutrient concentration in the hypolimnion zone and strong oxygen depletion just below the thermocline.

The results of our study confirm that the thermocline zone is favorable for phytoplankton. However, the maximum concentrations of phytoplankton were observed in the lower layer of the thermocline and upper part of the hypolimnion. Increased algal densities below the thermocline often were related to nutrient availability (Fee et al. 1996; Fasham et al. 1985), but in our study there was no significant increase of nutrient concentration in the metalimnion. Many studies emphasize the importance of algal sinking velocity for vertical distribution patterns (Jager et al. 2010). Higher density and viscosity of water in the thermocline zone limited sedimentation loss, which is especially important in nonmotile species (Diehl 2002). Due to sedimentation, algae tend to progressively accumulate at a depth where cell density equals water density (neutral buoyancy), or, at least, where water density increases (Wetzel 2001, Camacho 2006). Typically, deep chlorophyll layer is formed by only one or a few algal species, whose population densities are extremely high compared to epilimnetic algal abundance (Gasol et al. 1992; Miracle et al. 1992). Deep chlorophyll layers in our lakes were caused mostly by the diatoms and cryptophytes. Fast sinking algae like diatoms (heavy and nonmotile) could benefit from greater water density (Arvola et al. 1991; Reynolds 1992; Winder et al. 2009). We observed the maximum concentrations of diatoms in the metalimnion of all studies lakes, while maximum density of cryptophytes was observed below the thermocline.

The phytoplankton of Lake Hańcza and Lake Szurpiły was intensively studied (Spodniewska 1978; Hutorowicz and Napiórkowska-Krzebietke 2008; Grabowska et al. 2006; Jekatierynczuk-Rudczyk et al. 2012). The dominant species in 1999–2001 was a cyanophyte *Aphanocapsa incerta* (Hutorowicz and Napiórkowska-Krzebietke 2008). The diatoms of the genus *Cyclotella* were the most important group with domination of *Cyclotella radiosa* (Grunow) Lemmerm, 1900 (Jekatierynczuk-Rudczyk et al. 2012). Cryptophytes and chrysophytes are often very motile species and can have mixotrophic feeding strategies (Vincent and Goldman 1980). They can capitalize on higher nutrient levels and bacterial biomass present at the edge of the hypolimnion, because of their low light needs (Ptacnik et al. 2003). Deep chlorophyll layers formed by diatoms and cryptophytes have been reported for many lakes of North America and Europe (e.g., Jackson et al. 1990; Camacho et al. 2001; Stoermer et al. 1996; Camacho 2006). However, some authors reported deep chlorophyll maxima formed by cyanobacteria (e.g., Kasprzak et al. 2000) as well as other algae (e.g., Zvikas 2005).

The results of our studies have shown that different groups of algae reached maximum density at different depths using various niches. Green algae were found in the upper layers of the water, because they are less sensitive to light distribution (Litchman 2000). Diatoms and cryptophytes reached higher densities in lower water layers with maximum densities noted at different depths. Models of vertical phytoplankton distribution for poorly mixed water columns have shown that the phytoplankton maximum occurs at the depth for which they are equally limited by light and nutrients (Klausmeier and Litchman 2001). The models also revealed that the light:nutrient ratio is an important determinant of phytoplankton dynamics (Huisman and Weissing 1995; Diehl 2002).

Relative to the phytoplankton, the vertical distribution of the more motile zooplankton can be linked not only to abiotic forces (temperature, turbulence) but also to the distribution of their prey (phytoplankton) and predators (e.g., Leibold 1990; Pinel-Alloul 1995; Masson et al. 2004). The zooplankton in our study reached the highest density in the thermocline zone. Many of the previous studies showed that zooplankton migrate to the rich food source located in the metalimnion (Jürgens et al. 1994; Gasol et al. 1995; Adrian et al. 2001; Adrian and Schipolowski 2003; Lampert and Grey 2003; Francis et al. 2011). The food resources for zooplankton in deep-water layers can be as profitable as those from the upper layers (Winder et al. 2003), while these resources are much more abundant in the deep chlorophyll layer (Camacho 2006). *Cryptomonas*, which are common in deep-water layers, are generally considered as a high-quality food (Barone and Naselli-Flores 2003), and there are many evidences that *Cryptomonas* are a favorable food for the *Daphnia* species. We found that vertical distribution of dominant species, *D. cucullata* was strongly related with phytoplankton distribution. *D. cucullata* effectively migrated to the deeper zone and could accumulate metalimnetic production via grazing and translocate a large proportion of organic matter from the metalimnion into the water column (Brosseau et al. 2012), whereas smaller species like *Bosmina* spp. was not affected by the thermocline and vertical distribution of phytoplankton. This could be the evidence that food resources in low trophic lakes are the most important factor affecting vertical distribution of large-bodied daphnids (Dini and Carpenter 1991; Williamson et al. 2011). While the metalimnion offers a rich phytoplankton food source, energetically it may not be profitable to spend time feeding in cold waters where metabolic activity is constrained (Lampert and Grey 2003). Large-bodied species are also susceptible to visual predation (Carpenter and Kitchell 1993) and distribution of these species reflects a trade-off between avoiding predation and maximizing food consumption (Johnsen and Jakobsen 1987). However, some research revealed that *Daphnia* do not migrate to the metalimnion for feeding on metalimnetic carbon, but it is probably migrating deep enough during the day to avoid visual predation (Dini and Carpenter 1991, Brosseau et al. 2012).

Vertical distribution of rotifers is probably under the influence of many different factors, food conditions being among the most important. However, during a day, rotifers may move vertically to avoid both competitors and predators (Karabin and Ejsmont-Karabin 2005). Another factor influencing occurrence of small rotifers is the presence of large *Daphnia*, which are known to kill and rapidly exclude rotifers (Gilbert 1988a). This interference may affect the species structure of rotifers. However, in Lakes Hańcza and Szurpiły the highest biomass of Rotifera was noted at depths of the highest occurrence of large *Daphnia*. The explanation is that rotifer biomass was in these tow lakes built mostly by either colonies of *C. hippocrepis*, or by large predator *A. priodonta*, which are too large to be suppressed by *Daphnia* (Gilbert 1988b).

## Conclusions

Our study confirms significant change of hydrochemical and biological parameters in the metalimnion zones. The thermocline zone was a favorable place for plankton communities. However, the deep chlorophyll maxima were observed just below the thermocline and were caused by the diatoms and cryptophytes. Such phytoplankton can capitalize on higher nutrient levels and bacteria biomass present at the edge of the hypolimnion because of their tolerance for lower light than in the case for many other phytoplankton. The maximum concentrations of diatoms were found in the thermocline, where heavy and nonmotile algae could benefit from greater water density. While cryptophytes reaches maximum concentration in the upper hypolimnion because they are often very motile and have mixotrophic feeding strategies. Vertical distribution of large crustacean zooplankton was similar to the distribution of phytoplankton. Especially, *D. cucullata* were strongly related with the phytoplankton distribution and reached maximum densities in deep chlorophyll layers, and, conversely, smaller crustacean species and rotifers were not affected by the vertical distribution of phytoplankton.
